# Monoclonal Antibody Functionalized, and L-lysine α-Oxidase Loaded PEGylated-Chitosan Nanoparticle for HER2/Neu Targeted Breast Cancer Therapy

**DOI:** 10.3390/pharmaceutics14050927

**Published:** 2022-04-24

**Authors:** Kandasamy Saravanakumar, Anbazhagan Sathiyaseelan, Soyoung Park, Song-Rae Kim, Veeraraghavan Vishnu Priya, Myeong-Hyeon Wang

**Affiliations:** 1 Department of Bio Health Convergence, Kangwon National University, Chuncheon 200-701, Gangwon-do, Korea; saravana732@kangwon.ac.kr (K.S.); sathiyaseelan@kangwon.ac.kr (A.S.); 202116272@kangwon.ac.kr (S.P.); 2 Chuncheon Center, Korea Basic Science Institute (KBSI), Chuncheon 24341, Gangwon-do, Korea; ksr87@kbsi.re.kr; 3 Department of Biochemistry, Saveetha Institute of Medical and Technical Sciences, Saveetha Dental College, Saveetha University, Chennai 600077, India; vishnupriya@saveetha.com

**Keywords:** L-lysine α-oxidase, HER2+, trastuzumab, breast cancer, PEGylated chitosan

## Abstract

**Simple Summary:**

Breast cancer is one of the dominant cancers that threaten human beings worldwide. Moreover, the treatment of HER2+ breast cancer is challenging due to heterogeneity. The L-lysine α-oxidase (LO) enzyme is a well-known antitumor enzyme, but its clinical utility has been limited due to side effects, decreased stability, and inability to target tumor cells. To overcome the clinical challenges in delivery of LO enzymes and improve HER2+ breast cancer therapeutics, the present study developed the dual stimuli responsive nanocarrier system (CS-LO-PEG-HER NPs) for pH sensitive and HER2/neu targeted breast cancer therapy.

**Abstract:**

Herein, we designed a nanocarrier to deliver the LO specifically to HER2+ breast cancer (BC) cells, where functionalization of mAb (anti-HER2+) with PEGylated chitosan enabled it to target the HER2+ BC cells. Taking advantage of overexpression of HER2+ in cancer cells, our nanocarrier (CS-LO-PEG-HER NPs) exhibited promising potency and selectivity against HER2+ BC cells (BT474). The CS-LO-PEG-HER NPs demonstrated the cytotoxicity in BT474 cells by promoting reactive oxygen species, mitochondrial membrane potential loss, and nucleus damage. The biocompatibility of CS-LO-PEG-HER NPs was evidenced by the hemolysis assay and H & E staining of major organs. The CS-LO-PEG-HER NPs showed anticancer potency against the BT474-xenograft tumor-bearing mice, as evident by the reduction of tumor size and cell density. These results indicate that CS-LO-PEG-HER NPs are biocompatible with mice while inhibiting tumor growth through alter the oxidative stress. Overall, this work provides a promising approach for the delivery of LO for good therapeutic effect in combination with mAb.

## 1. Introduction

L-lysine α-oxidase (LO) was first discovered in *Trichoderma viride*, then in *T. harzianum* Rifai, in the 1980s [1,2]. This enzyme has been shown to inhibit the proliferation of cancer cells, such as human lung squamous cell carcinoma (RERF-LC-AI), prostate cancer (PC3), cervical cancer cell line (HeLa), ovarian cancer (SKOV3), breast cancer (MCF7), colon cancers (LS174T and HT29) and erythromyeloblastoid leukemia (K562), without causing any significant damage to normal human umbilical vein endothelial cells (HUVECs) [3,4]. Breast and ovarian cancer cells are more sensitive to LO than others [5]. The enzyme LO triggers H_2_O_2_ generation, which causes cytotoxicity through damaging the DNA and mitochondrial membrane [6]. Moreover, the cytotoxicity of LO is dependent on the concentration of L-lysine in the tissue [5]. The enzyme LO exhibits high antitumor activity due to the higher elevation of L-lysine in tumor tissue compared to normal tissue [4,7]. L-Lysine is an essential amino acid present in body tissue and blood plasma, which plays an important functional role in metabolism [8]. Therefore, deprivation of L-lysine is an important issue caused by LO administration to animals. Although LO is efficient in antitumor activity, an administration of 150 U/kg at a 48 h interval might cause toxicity, weight loss (>30%) and loss of L-lysine in blood plasma, which results in the death of mice [3]. The LO administration (75 U/Kg of four doses for 10 days at 2 days interval) suppresses the growth of cancer cells in Balb/c nude mice that were transplanted with cancer cells [5]. 

The LO is well known as a potential antitumor enzyme, but its overdose can cause toxicity to L-lysine in normal cells. Further, the therapeutic efficacy of LO is largely limited due to degradation and insufficient discharge at the cancer site. There is demand for the development of new strategies to release LO at cancerous sites for enhanced cancer therapy without causing any adverse effects to normal cells. Similarly, the overexpression of the human estrogen receptor (HER2+) efficiently stimulates breast cancer (BC) growth. Therefore, blocking the signal transfer between HER2 receptors would inhibit the proliferation of BC. Thus, trastuzumab (Herceptin) monoclonal antibody (mAb) is used to treat breast cancer by targeting the HER2/neu receptor [9]. In nanotechnology, herceptin (ligand) has gained attention for HER2+ BC targeted drug delivery due to its biocompatibility, safety, and high efficiency in binding to HER2+ receptors in BC. Although several works have reported the combination of drug and mAb delivery for enhanced cancer therapy, there is none of the work reported on antitumor enzyme delivery associated with mAb.

In this context, a nano-drug delivery system using chitosan and polyethylene glycol has the promise to release various drugs in the cancer microenvironment based on pH [10,11,12]. The efficacy of therapeutic enzymes is known to be increased by conjugation with antibody, folic acid, daidzein, liposomal nanoparticles, chitosan nanoparticles, gold nanoparticles, dendrimer, and nanotubes [13,14,15,16,17,18]. In addition, the chitosan nanoparticles are known to deliver monoclonal antibody, genes, and anti-cancer enzymes in the cancer sites [19,20,21]. To overcome the clinical challenges of higher toxicity and instability in the LO enzyme and increase its therapeutic efficacy, the present study was hypothesized to develop a PEGylated chitosan-based enzyme (LO) delivery system for HER2/neu targeted breast cancer therapy. A pH-dependent and herceptin (trastuzumab) functionalized L-lysine α-oxidase loaded PEGylated chitosan nanoparticle was engineered and characterized for HER2/neu targeted breast cancer therapy.

## 2. Materials and Methods

### 2.1. Chemicals, Cell Line, and Animals 

Chitosan (CS; CAS No. 9012-76-4, low molecular weight −50,000–190,000 Da; 75–85% deacetylated chitin, poly (D-glucosamine), polyethylene glycol (PEG 6000), L-lysine α-oxidase (LO) from *Trichoderma viride*, ethidium bromide (EB), acridine orange (AO), hydrochloric acid, 1-ethyl-3-(3-dimethylaminopropyl) carbodiimide hydrochloride (EDC: HCl), rhodamine 123 (Rh123), N-hydroxysuccinimide (NHS), propodium iodine (PI), dichlorofluorescein diacetate (DCFH-DA) were purchased from Sigma-Aldrich (St. Louis, MO, USA). Antibody anti-HER2 (Herceptin, trastuzumab; A1046-100 Lot. 4E31A10460) was obtained from BioVision Inc., Milpitas, CA, USA. The cell viability assay kit CELLO MAXTM was procured from MediFab, Republic of Korea. Cell culture media (Roswell Park Memorial Institute Medium; RPMI), fetal bovine serum (FBS), SnakeSkin™ Dialysis Tubing (10 K MWCO, 35 mm), penicillin and streptomycin (P&S), and apoptosis kit (Annexin V FITC and PI) for flow cytometry were acquired from ThermoFisher Scientific (Waltham, MA, USA). Human breast cancer cell line (BT474) was purchased from Korean Cell Line Bank, (KCLB), Seoul, Korea. Seven-week-old BALB/c/nu/nu female mice weighing 20–22 g were purchased from Nara Biotech, Seoul, Korea. 

### 2.2. Preparation of CS-LO NPs/CS-LO-PEG NPs

The characteristics of the chitosan used in the present study are reported in our previous work [22]. The CS-LO NPs were prepared by the TPP-ionotropic gelation method [23]. In brief, 5 mg of CS was dissolved in 10 mL of 0.5% (*v*/*v*) acetic acid and stirred at 50 °C for 4 h. The TPP solution (1 mg/mL) was prepared in deionized (DI) water and stirred for 2 h. The CS and TPP solutions were filtered through a syringe filter (0.45 µm). The pH of the CS solution was adjusted to 5.0 using 0.2 M NaOH. The 25 mL of TPP (1 mg/mL) was added to the 75 mL of CS (0.5 mg/mL) solution drop by drop and stirred at room temperature for 4 h. PEGylated CS nanoparticles were prepared according to the method reported earlier with slight modifications [24]. A total of 10 mg of the PEG 6000 was dissolved in CS (0.5 mg/mL) solution under stirrer condition for 1 h, and then the pH of the PEG-CS solution was adjusted to 5.0 using 0.2 M NaOH. Afterwards, the TPP (1 mg/mL) solution was added to the CS-PEG solution to form the nanoparticles. The different concentrations of LO (0.1–1 mg/mL) were incorporated into the CS solution or CS-PEG solution before the addition of TPP. The CS-LO NPs and CS-LO-PEG NPs were centrifuged at 14,000 rpm. The pellets were lyophilized and preserved at 4 °C in a refrigerator. 

### 2.3. Functionalization of Antibody Anti-HER2 (Trastuzumab) in CS-LO-PEG NPs

The functionalization antibody in the NPs was carried out through covalent bonding performed according to the method reported elsewhere [25]. The COOH group of CS-LO-PEG NPs was activated by the addition of 10 µL of NHS (10 mM) and EDC (10 mM) and then added to CS-LO-PEG NPs (0.5 mg/2 mL) stirring at 150 rpm at ambient temperature for 8 h. Afterwards, the anti-HER2 (trastuzumab) (100 µg/mL of COOH group activated CS-LO-PEG NPs) was added and kept stirred using the magnetic stirrer at ambient temperature for 12 h. Then the anti-HER functionalized CS-LO-PEG NPs were named CS-LO-PEG-HER NPs and dialyzed using the SnakeSkin™ Dialysis Tubing (10 K MWCO, 35 mm) in phosphate buffer for 4 h, and then centrifuged at 20,000 rpm for 2 min, and the pelleted nanoparticles were stored at 4 °C for characterization, drug release, and anticancer experiments. 

### 2.4. Enzyme Assay

Total protein was determined using the Bradford reagent (Bio–Rad). The enzyme (LO) was determined by measuring the H_2_O_2_ production in the reaction mixture at 555 nm using a spectrophotometer (SpectraMAx@ Plus 384 Microplate reader, Molecular devices) [5,26]. In brief, the enzyme solution was added to trisphosphate buffer (20 mM; pH 8.0) containing 2 mM of o-dianisidine, peroxidase (5 µg/mL), and substrate L-lysine (2 mM) and incubated at 30 °C for 5 min. Then absorbance was measured at 555 nm and the enzyme activity was expressed as IU [26]. 

### 2.5. Enzyme Loading and Entrapment Assay

Different concentrations of LO (0.1–1 mg/mL) were loaded onto CS-PEG-NPs. After completion of the LO loading producers, the unloaded LO was collected by centrifugation of the LO-CS-PEG NPs suspension at 12,000 rpm for 20 min. The supernatant was used as the LO source for the enzyme assay described in Section 2.4. The enzyme present in the supernatant was measured and subjected to the following equations to calculate the enzyme (LO) entrapment and loading efficiency: EEE (%) = (Ei − Ef)/Ei, where Ei: total input of LO, Ef is the amount of unloaded LO in the supernatant; and ELE = (Ei − Ef)/M, where Ei is the total amount of LO input, Ef is the amount of unloaded LO in the supernatant, and M: total weight of nanoparticle (LO-CS-PEG NPs). 

### 2.6. Characterization of Nanoparticles 

A series of analytical methods were used to confirm the successful formation of CS-LO-PEG-HER NPs. The loading of LO and, functionalization of mAb (Anti-HER) were analyzed by FTIR (Fourier-transform infrared spectroscopy; PerkinElmer Paragon 500, Waltham, MA, USA) and NMR (Nuclear Magnetic Resonance; FT-NMR, Bruker, 600 MHz, MA, USA). For the FTIR analysis, the nanoparticle samples, including CS, PEG6000, LO, HER, CS-LO NPs, CS-LO-PEG NPs, and CS-LO-PEG-HER NPs, were prepared as KBr pellets using the standard sample preparation method, and the IR scanning was performed from the 400–4000 cm^−1^. To determine the size and zeta potential of nanoparticles, DLS (Dynamic light scattering; zeta potential particle size analyzer Malvern, Eindhoven, The Netherland) and ELS (Electrophoretic light scattering) analysis were used. For the DLS/ELS analysis, 10 µg of each NPs was dissolved in 3 mL of DI water, sonicated for 2 min, and filtered using a 2 µm syringe filter. The filtered nanoparticle suspension was used for DLS analysis. Transmission electron microscopic (JEOL-JSM 1200EX, Tokyo, Japan) analysis was used to observe the morphology of nanoparticles. For TEM analysis, 10 µg of each NPs was dissolved in 1 mL of ethanol, sonicated for 2 min, and negative stained with 2% uranyl acetate then loaded into a copper grid for TEM observation [27]. 

### 2.7. In Vitro Enzyme Release Assay 

In vitro release of the enzyme LO from the CS-LO-PEG-HER NPs was determined at three different pH values (5.0, 6.8, and 7.4). In brief, the 5 mg of the CS-LO-PEG-HER NPs were dissolved in 5 mL of different pH buffer solutions. The nanoparticle suspension was loaded in SnakeSkin™ Dialysis Tubing (35 mm) membrane bag. The nanoparticle loaded membrane was kept in a 250 mL beaker containing the 50 mL dissolution buffer solution (pH 5.0, 6.8, and 7.4) and allowed for magnetic stirring (50 rpm). Aliquots of 5 mL were collected at different time intervals (0–70 h) by replacing the same volume of dissolution buffer, and the enzyme release was measured using the enzyme assay methods described in Section 2.5. The experiments were performed three times, and pH-dependent release of the LO was calculated and expressed as the cumulative release (%).

### 2.8. Cell Culture and Blood Compatibility Assay 

#### 2.8.1. Cellular Internalization 

The effect of the anti-HER functionalization in nanoparticles (CS-LO-PEG-HER NPs and CS-LO-PEG NPs) on cellular internalization was analyzed by in vitro cell culture experiments. In brief, the BT474 cells (1 × 10^4^) were cultured in a 5 mL of RPMI medium incorporated with 10% of FBS and 1% antibiotic solution in six-well plates (costar) in 5% CO_2_ atmosphere at 37 °C for 24 h. Afterward, 50 ng/mL of each nanoparticle (CS-LO-PEG-HER NPs and CS-LO-PEG NPs) was treated for 12 h, then the cells were collected using the aseptic cell scraper, dehydrated, and observed under TEM. 

#### 2.8.2. Hemolysis Assay

The blood compatibility of the nanoparticles (CS-LO-PEG-HER NPs and CS-LO-PEG NPs) was tested in sheep blood (Carlina, Seoul, Korea). In brief, 1 mL of sheep blood was dissolved in 10 mL of PBS (pH 7.4) and centrifuged at 2000 rpm for 10 min at 4 °C. After the centrifugation, the pellet containing the RBC was collected by washing three times with PBS (pH 7.4). The RBC suspension (4% *v*/*v*) was prepared in PBS for the hemolysis assay. For the assay, 200 µL of RBC suspension and 400 µL of different concentrations of each nanoparticle (12.5, 25, 50, 100, 150, 200 ng/mL) were added and incubated at 37 °C for 1 h and then centrifuged at 2000 rpm at 4 °C for 10 min. The absorbance of the supernatant was observed at 545 nm using the UV spectrophotometer. Here, 1% (*v*/*v*) of triton X-100 was used as a negative control while the PBS was used as a blank. The percentage of hemolysis was calculated by applying the formula: hemolysis (%) = (test/control) × 100. 

#### 2.8.3. Cytotoxicity 

The cytotoxic effect of nanoparticles, enzymes, and antibody (anti-HER2) treatments in BT474 cells was analyzed by the WST assay. In detail, the BT474 cells (1 × 10^4^) seeded in the 96 well plates containing the 100 µL of RBMI media (10% FBS and 1% antibiotic solution) and cultured at 37 °C at a 5% CO_2_ atmosphere for 24 h. Then different concentrations (25, 50, 100, 150, 200 ng/mL) of nanoparticles, enzyme, and antibody (anti-HER2) were treated at the same incubation condition for 12 h. After the treatment, 10 µL of WST solution was added and incubated for 45 min. The absorbance was read at 450 nm using the UV spectrophotometer. The cell viability (%) was calculated using the formula (OD of test/OD control) × 100.

#### 2.8.4. Fluorescent Microscopic Assay

Effect of the nanoparticles, enzyme, and antibody (anti-HER2) treatments were determined in BT474 cells on mitochondrial membrane potential (MMP), nucleus, and reactive oxygen species (ROS) generation. In brief, the BT474 cells were cultured as mentioned in Section 2.8.1. After the confluence, the IC50 concentration of each sample was treated at 37 °C, in a 5% CO_2_ atmosphere for 12 h. Then the MMP changes were observed by staining with Rh123, and the nucleus damage was visualized using DAPI staining. The ROS generation was noted through DCFH-DA staining, while the apoptosis stages were observed by AO/EB staining. All stain preparations and staining producers were carried out in accordance with the methods described in our previous works [28,29]. 

#### 2.8.5. FITC, Annexin V Apoptosis Assay 

The treatments induced apoptosis stages (live, early, late apoptosis, and necrosis) in BT474 cells were measured by flow cytometer using Annexin V, FITC staining according to the protocols described in the manufacture’s instruction. In brief, the BT474 cells were cultured as mentioned in Section 2.8.1. The IC50 samples were treated for 12 h, and cells were collected using the aseptic cell scraper and washed with PBS (pH 7.4) by centrifugation at 4 °C for 4 min. Then the cells were fixed with 100 µL of binding buffer (1×) for 10 min at ambient temperature. Afterward, 1 µL of PI and 5µL of FITC-labeled annexin V were added and incubated for 15 min at room temperature. Finally, 400 µL of binding buffer (1×) was added, filtered, and analyzed using the flow cytometer (BD FACS aria II). 

#### 2.8.6. Cell Cycle Assay

The effect of nanoparticles, enzymes, and antibody (anti-HER2) treatments on the cell cycle of BT474 cells was analyzed by PI staining using a flow cytometer. The PBS was used to prepare the 70% ethanol and PI stocks. For the PI stock, 50 µg of PI and 100 µg of RNase A were dissolved in 1 mL of PBS (pH 7.2). The above-mentioned samples of treated cells were collected as described in Section 2.8.1 and fixed in 70% ethanol for 10 min. Then the cells were washed with PBS by centrifugation at 2000 rpm for 4 min at 4 °C. An equal volume of each treatment was adjusted using a hemocytometer and then stained with 500 µL of PI stock solution for 30 min. The cell cycle arrest was analyzed using the flow cytometer (BD FACS aria II). 

### 2.9. In Vivo Animal Model

Seven-week-old athymic BALB/c female mice (20–22 g) were purchased from Nara Biotech, Seoul, Korea. Animal studies were performed according to the Kangwon National University Ethical Policy (KW-201019-1). The mice were housed (6 mice/cage) at a 12/12 h light/dark cycle and fed with a standard diet and water ad libitum. The BT-474 cells (1 × 10^6^/mice) were subcutaneously injected into the right flank. The 0.1 mL of PBS was used as a cell vehicle and the implanted cells were allowed to grow for 10 days. Once tumor size reached 100–160 mm^3^, the mice were randomly grouped for treatments, and each treatment group contained 4 mice, which is followed according to the Kangwon National University Ethical Policy: (i) saline water (PBS), (ii) LO (200 µg/kg), (iii) HER (200 µg/kg), (iv) CS-LO-PEG NPs (200 µg/kg), and (v) CS-LO-PEG-HER NPs (200 µg/kg). These samples were injected intravenously once a day for 12 days. During the experiment, the body weight and tumor volume were measured using the laboratory weighing balance and Vernier caliper. After the treatments, the mice were sacrificed by cervical dislocation, and organs and tumors were collected for histopathological analysis using Hematoxylin and Eosin staining according to the method reported earlier [27,30]. 

### 2.10. Statistical Analysis 

Each experiment was repeated at least three times, and the results are expressed as a mean ± standard error. The significance of the factor was determined using analysis of variance (ANOVA) and a post-hoc test (Duncan’s test). All statistical analysis is carried out via the use of a virtual tool (IBM SPSS statistical software version 20; IBM, New York NY, USA).

## 3. Results and Discussion 

### 3.1. Synthesis and Characterization 

#### 3.1.1. DLS, Enzyme Entrapment, Loading Efficiency 

The present study designed a nano-drug delivery system to deliver the LO specifically to HER2+ breast cancer (BC) cells, where functionalization of mAb (anti-HER2+) with PEGylated-chitosan enabled the targeting of the HER2+ BC cells. The physicochemical properties of NPs in response to the input of LO are shown in Table 1. The results revealed that the LO input significantly influenced the average size, zeta potential, polydispersity index (PDI), enzyme entrapment efficiency (EEE), and enzyme loading efficiency (ELF) (Table 1). Although these indicators varied in response to the LO input, all the nanoparticles exhibited the amicable properties that are favorable for biomedical drug delivery. Further, the size (138.53–182.60 nm) in line with PDI was 0.12–0.44, and the zeta potential was 26.73–36.60 mV (Table 1). The size of the nanoparticles has a vital role in cellular internalization and uptake of nanomedicine, as indicated by earlier reports [31], and the nanoparticles size of <200 nm is known to enhance cellular uptake and internalization through the EPR (permeability and retention) effect [32]. The results of the present work recorded that the input of 0.5 mg of LO had achieved CS-LO-PEG NPs with a size of 144.1 ± 2.96 nm with zeta potential of 35.86 ± 1.33 mV, and PDI of 0.28 ± 0.03, and it also exhibited the optimal EEE of 59.15 ± 0.44%, ELE of 14.26 ± 0.85%. These characteristics of nanoparticles are favorable for clinical application. Hence, they were selected for further characterization and anti-HER functionalization.

The DLS and ELS were again performed to show the zeta size, zeta potential, and PDI of CS-LO NPs, CS-LO-PEG NP, CS-LO-PEG-HER NPs (Figure S1). The results indicated that the size of the NPs increased with PEGylating and anti-HER2 functionalization. The CS-LO NPs exhibited a z-average size of 137.0 ± 1.02 nm, and a zeta potential of 36.4 ± 2.18 mV with PDI of 0.360 ± 0.04 (Figure S1a,b). The CS-LO-PEG NP displayed a z-average size of 143.0 ± 0.98 nm, and a zeta potential of 35.3 ± 0.52 mV with PDI of 0.357 ± 0.02 (Figure S1c,d), whereas CS-LO-PEG-HER NPs unveiled a z-average size of 165.9 ± 1.02 nm, and a zeta potential of 34.2 ± 1.60 mV with PDI of 0.300 ± 0.12 (Figure S1e,f). The results revealed that the 0.5 mg of LO-loaded nanoparticles (CS-LO NPs, CS-LO-PEG NP, and CS-LO-PEG-HER NPs) synthesized in the present work unveiled the optimal size, zeta potential, and PDI for drug delivery. The PDI value of a nanoparticle of <0.5 and a zeta potential of >30 mV indicates the mono-dispersion and stability of the NPs [33]. Furthermore, the positive charge of the NPs improved electrostatic interaction, cellular penetration, and uptake with the negatively charged cell membrane [34]. 

#### 3.1.2. Morphological Characterization by TEM

**Figure 1:**
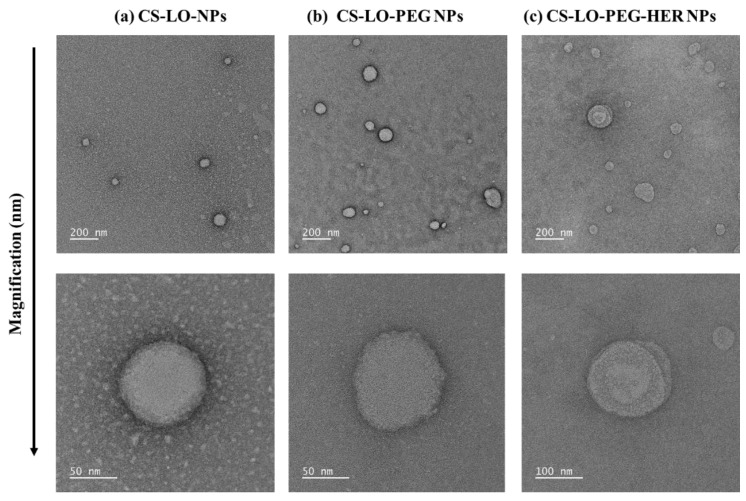
Transmission electron microscopic observation of HER functionalized L-lysine α-oxidase loaded chitosan-polyethylene glycol (PEG) nanoparticles. CS-LO NPs (**a**), CS-LO-PEG NPs (**b**) and CS-LO-PEG-HER NPs (**c**). Where CS is chitosan, LO is L-lysine α-oxidase, PEG is Polyethylene glycol 600, HER is Herceptin (Trastuzumab).

The TEM results of CS-LO NPs, CS-LO-PEG NPs, and CS-LO-PEG-HER NPs are shown in Figure 1a–c. The morphology of the nanoparticles was found to be spherical and homogeneous spheres with size of <100 nm, which is smaller than the DLS measurement of size. The size difference between TEM and DLS was observed due to the differences in the sample preparation and hydrodynamic properties of NPs [22,35]. In addition, the DLS was performed to analyze the size of the particle in a liquid state according to the Stokes–Einstein principle, while TEM principally works in the dry state of samples under high vacuum pressure [36].

#### 3.1.3. Surface Chemistry Analysis 

**Figure 2:**
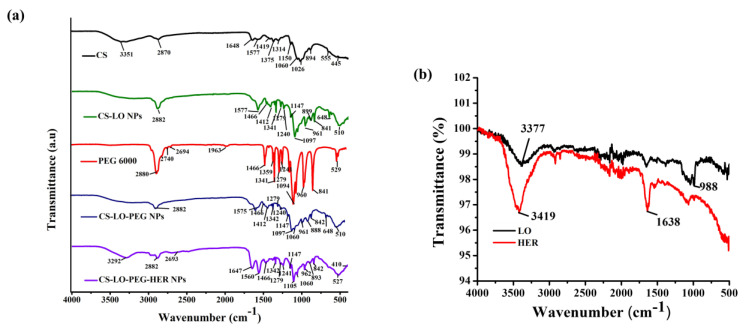
FTIR analysis of herceptin (trastuzumab) functionalized L-lysine α-oxidase loaded chitosan-polyethylene glycol (PEG) nanoparticles. Functional group changes in CS, CS-LO NPs, PEG600, CS-LO-PEG NPs and CS-LO-PEG-HER NPs are shown in (**a**), while LO and HER are shown in (**b**).

The FTIR facilitated the analysis of the formation, loading, and functionalization of the LO and antibody (anti-HER2+) in the CS-LO-PEG-HER NPs in comparison with CS, PEG, HER, LO, and its conjugated NPs. To verify the loading of the LO, and functionalization of anti-HER, the IR spectrum of free LO, HER, CS-LO NPs, CS-LO PEG NPs were compared, and the results are shown in Figure 2a,b. The CS exhibited the characteristic vibration at 3351 cm^−1^, 1648 cm^−1^, and 1577 cm^−1^ assigned to the O-H stretching, C=O, amine I, and amine II respectively, while the 1419 cm^−1^, 1375 cm^−1^, 1314 cm^−1^ corresponding to O-H, and other characteristics peaks at 1060, 1026 assigned for C-N amine while peaks including 894 cm^−1^, 555 cm^−1^, 445 cm^−1^ accounted for C-H bending of CS [37,38]. The IR spectrum of the LO displayed primary protein absorption characteristic peaks at 3377 cm^−1^ accounting for N-H (primary amine), while 988 corresponded to C=C bending [39], Similarly, the protein absorption peaks for the lipase B and horseradish peroxidase are reported in the earlier work by using the IR spectrum [38,40,41]. The CS-LO NPs showed the overlapping and reappearance of characteristic peaks of CS and LO at 2882 cm^−1^, 1577 cm^−1^, and 961 cm^−1^, respectively. Besides, some of the peaks related to the LO changed due to the interaction of amino groups with polymer surfaces [40]. In addition, the peaks of 1240 cm^−1^ and 899 cm^−1^ in CS-LO NPs indicate the formation of P=O and P-O-P bonds related to the interaction of an amino group of CS and the phosphate group of TPP mediated CS NPs formation [38,42]. 

The PEG 6000 illustrated the characteristic peaks at 2880 cm^−1^, 2740 cm^−1^, 2694 cm^−1^, corresponding to C-H, and 1466 cm^−1^ and 960 cm^−1^ accounted for by the ether bond [38]. The CS-LO PEG NPs exhibited the overlapping of the characteristic peaks, CS, LO and PEG, specifically the peaks such as 2882 cm^−1^, 1575 cm^−1^, 1466 cm^−1^, 961 cm^−1^ that reappeared in the IR spectrum of CS-LO PEG NPs. Furthermore, the peaks that appeared at 2882 cm^−1^, 1147 cm^−1^ and 1097 cm^−1^ evidenced the successful PEGylation with the amine group of CS and the formation of CS-LO PEG NPs [43]. The free anti-HER exhibited the peaks at 3419 cm^−1^ and 1638 cm^−1^, which corresponded to the primary (N-H stretching) and secondary amine (N-H bending) groups [39]. The CS-LO-PEG-HER NPs showed the reappearance or overlapping of the IR peaks corresponding to CS (3292 cm^−1^), LO (962 cm^−1^), PEG (2693 cm^−1^), HER (1647 cm^−1^) with minor shifting from the original appearance, and this indicated the successful formation of CS-LO-PEG-HER NPs through TPP, EDC, and NHC mediated synthesis. 

### 3.2. In-Vitro Experiments 

#### 3.2.1. Drug Release and Intracellular Distribution 

**Figure 3:**
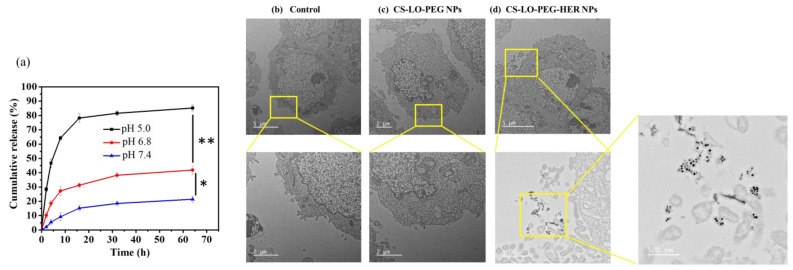
In vitro pH responsive enzyme release from the herceptin (trastuzumab) functionalized L-lysine α-oxidase loaded chitosan-polyethylene glycol (PEG) nanoparticles (CS-LO-PEG-HER NPs; (**a**)), cellular internalization without herceptin functionalized (CS-LO-PEG NPs) and herceptin functionalized (CS-LO-PEG-HER NPs) L-lysine α-oxidase loaded chitosan-polyethylene glycol (PEG) nanoparticles. (**b**–**d**) shown the nanoparticles internalization in high magnification. ** *p* < 0.01; * *p* < 0.05.

The stability and pH-responsive release of LO from CS-LO-PEG-HER NPs were tested in three different pH buffer solutions (pH 5.0, 6.8, and 7.4). The results indicated that the LO release significantly varied with the time and pH value (*p* < 0.05), and the higher release of >80% was found at pH 5.0, while the lower ~15 was found at pH 7.4 after 64 h (Figure 3a). The pH-responsive diffusion and degradation properties of CS and PEG triggered the LO release at pH 5.0. Similarly, several works have been reported the pH responsive release of drug from PEGylated CS [44,45]. The pH of the cancer microenvironment is reported to be pH ~5.2–5.4 [46]. Furthermore, the enzyme LO is stable in a wide range of pH and temperature [4]. 

Cellular penetration, and uptake are considered important factors for successful nanomedicine. Therefore, we experimented to compare the effects of dual stimuli (pH, and electrostatic interaction) and multi stimuli (electrostatic, ligand, and antibody interaction and pH) on cellular uptake and penetration of NPs by TEM. The results are shown in Figure 3b–d. The data confirmed the higher cellular uptake and distribution of CS-LO-PEG HER NPs than CS-LO PEG NPs. These results indicated that the Anti-HER2 (antibody–ligand) and HER2 (receptor) interaction triggered the higher cellular binding of formal CS-LO-PEG HER NPs than the bare CS-LO PEG NPs. 

#### 3.2.2. Blood Compatibility

**Figure 4:**
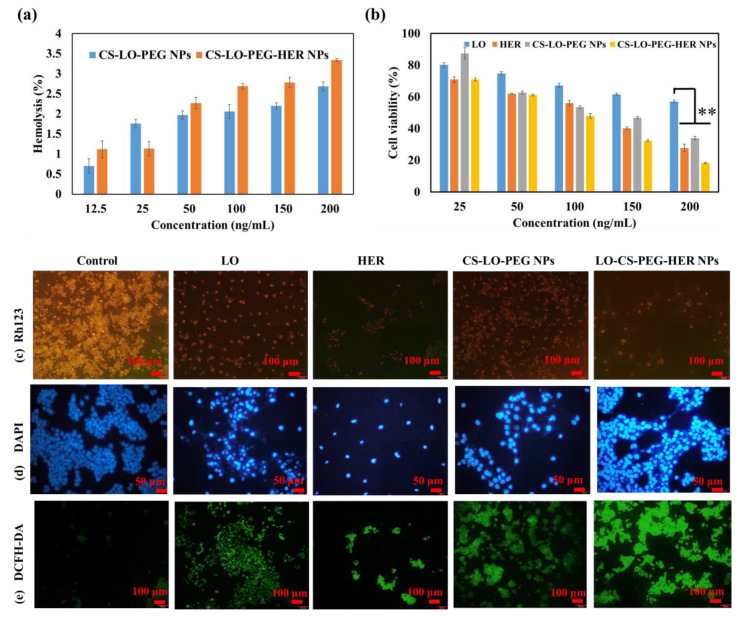
Blood compatibility of CS-LO-PEG-HER NPs and CS-LO-PEG- NPs (**a**), cytotoxicity of enzyme loaded nanoparticles in BT474 cell line (**b**), microscopic fluorescent analysis of enzyme or enzyme loaded nanoparticles induced mitochondrial membrane loss by Rh123 (**c**), nucleus damage by DAPI (**d**), reactive oxygen species generation by DCFH-DA (**e**). Where CS is chitosan, LO is L-lysine α-oxidase, PEG is polyethylene glycol 600, HER is herceptin (Trastuzumab). ** *p* < 0.01.

Nanomedicines are favorable to intravenous injection for the efficient delivery of drugs without any toxicity to normal cells [47]. In addition, the enzyme LO is known to cause the toxicity of L-lysine in blood plasma [3]. To verify the blood toxicity of LO, a hemolysis assay was performed and compared the activity of CS-LO-PEG HER NPs and CS-LO PEG NPs. As shown in Figure 4a and Figure S2, both NPs exhibited a hemolysis rate of <5% even at the high concentration of 200 ng/mL, and hence, CS-LO-PEG HER NPs and CS-LO PEG NPs did not cause obvious RBC lysis, as shown in Figure S2. It has been reported that <5% of hemolysis can be considered blood compatible and used for drug delivery applications [48,49].

#### 3.2.3. Cytotoxicity 

The WST assay was applied to investigate the comparative cytotoxicity effects of LO, HER, CS-LO-PEG NPs and CS-LO-PEG HER-NPs in BT474 cells (Figure 4b). The cytotoxicity significantly varied between the samples or concentrations. The CS-LO-PEG HER-NPs induced higher cytotoxicity than the other samples. This higher cytotoxicity of CS-LO-PEG HER-NPs was attributed to the increased cellular uptake through the interaction of anti-HER2 (ligand) and surface receptor (HER2+). According to earlier work, the functionalization of anti-HER2 (trastuzumab) would support the greater uptake of the nanomedicine through receptor-mediated endocytosis [50,51,52]. The overexpression of HER2+ in the breast cancer cell line (BT474) enabled the higher interaction and bonding between the mAb (anti-HER2) and receptor (HER2+), which led to more efficient cytotoxicity than the other samples, including LO, HER, and CS-LO-PEG NPs. This finding is also supported by the cellular uptake and internalization studies. 

#### 3.2.4. Fluorescent Staining Assay

**Figure 5:**
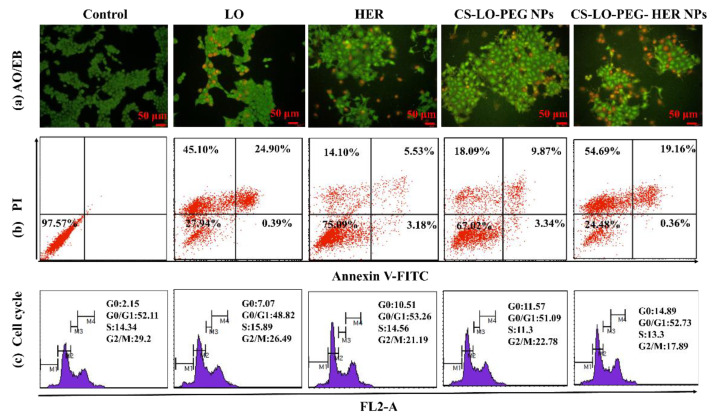
Effect of L-lysine α-oxidase, herceptin, and different nanoparticles treatment on apoptosis stages of BT474 cell line observed by AO/EB staining (**a**), FITC and annexin V staining based flow cytometry assay (**b**) and cell cycle stages by PI staining (**c**). Where CS is chitosan, LO is L-lysine α-oxidase, PEG is polyethylene glycol 600, HER is herceptin (Trastuzumab).

The cytotoxic effect of LO, HER, CS-LO-PEG NPs, and CS-LO-PEG HER-NPs in BT474 was further validated through a fluorescent staining assay (Figure 4c–e) Rh123 staining results revealed that the treatment of LO-CS-PEG-HER-NPs, HER, and LO caused a complete MMP loss, while CS-LO-PEG NPs exhibited moderate MMP loss as compared to control cells (Figure 4c). The loss of MMP was indicated through the loss of fluorescent intensity [53]. The nuclear stain DAPI is used to study the nucleus damage (nuclear fragmentation, chromatin condensation, margination). Moreover, DAPI would bind strongly with damaged cells as compared to healthy cells [54]. The results from DAPI staining revealed that the treatment of CS-LO-PEG HER-NPs caused higher cellular damage in the BT474 cells than in other samples (Figure 4d). The DCFH-DA is a dye used to measure the ROS generation under the excitation of fluorescents. The higher ROS generation was found in a decreasing order of sample treatments: LO-CS-PEG-HER NPs > CS-LO-PEG NPs > LO > HER (Figure 4e). These results agree with the general principle of LO-mediated cell toxicity through the production of H_2_O_2_ mediated oxidative stress [6]. Following this, the dual stain AO/EB assay was performed to observe the LO, HER, CS-LO-PEG NPs and CS-LO-PEG HER-NPs induced apoptosis stage in BT474 cells. The results showed more apoptosis (orange, chromatin damage) and necrosis (red, fragmented) cells than in other samples of treated cells (Figure 5a).

#### 3.2.5. Measurement of Apoptosis and Cell Cycle Arrest by Flow Cytometer 

A FITC annexin V, PI stain-based flow cytometry assay was executed to investigate the effect of LO, HER, CS-LO-PEG NPs, and CS-LO-PEG HER-NPs treatments in BT474 on apoptosis and cell cycle arrest (Figure 5b). Figure 5b displays the live cells in the left lower quadrants, early apoptosis in the right lower quadrants, and late apoptosis in the right upper quadrants, while the left upper quadrants showed the necrosis cells. The apoptosis or necrosis cells (%) were found higher in CS-LO-PEG HER-NPs (74.21%) and LO (70.39%) treated cells than in other treatments. Next, the cell cycle distributions due to the treatments in BT474 cells were investigated. The treatment of LO, HER, CS-LO-PEG NPs, and CS-LO-PEG HER-NPs showed a typical cell distribution pattern of G0, G0/G1, S, and G2/M phases in the cell cycle. The number of cells in each cell cycle stage significantly varied between the treatments (*p* < 0.05; Figure 5c). 

The treated cells that exhibited higher cell populations in the G0/G1 phase were LO (48.82%), HER (53.26%), CS-LO-PEG NPs (51.09%), and CS-LO-PEG HER-NPs (52.73%). Moreover, the cell population was found high in the G0 phase (apoptosis) of the CS-LO-PEG-HER NPs (14.89%) compared to the control untreated cells. The present results revealed that the treatment of CS-LO-PEG-HER NPs exhibited cell arrest in the G0/G1 phase. Similarly, earlier work reported that the cell population in the G0 phase was related to the apoptosis cells [55]. Cell cycle arrest and apoptosis are regular processes to inhibit the abnormal growth of cells through activating signals related to the apoptosis-related pathway [56]. 

### 3.3. In Vivo Experiments

#### 3.3.1. Survival, Tumor Size, and Body Weight 

**Figure 6:**
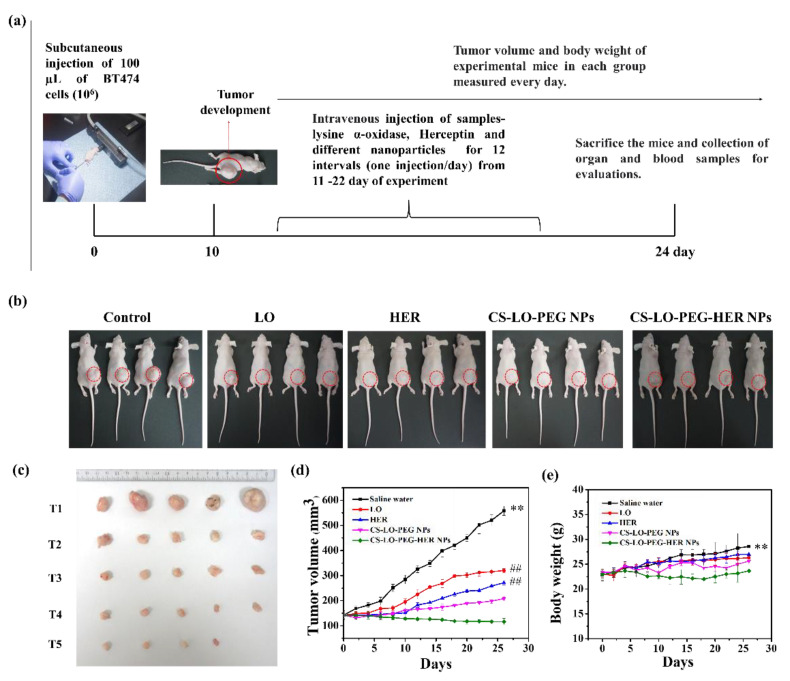
Effect of L-lysine α-oxidase, herceptin and different nanoparticles treatment on tumor bearing mice. Schematic illustration of in vivo antitumor study plan (**a**). Photographic visualization of tumor development (**b**), measurement of tumor volume in 24th day (**c**), tumor volume variation in relation to days (**d**) and body weight response to days (**e**). Where CS is chitosan, LO is L-lysine α-oxidase, PEG is polyethylene glycol 600, HER is herceptin (Trastuzumab). (T1) saline water (PBS), (T2) LO (200 µg/kg), (T3) HER (200 µg/kg), (T4) CS-LO-PEG NPs (200 µg/kg), and (T5) CS-LO-PEG-HER NPs (200 µg/kg). ** indicated the *p* < 0.01 significant variations between the untreated (saline water) mice with all other treated groups. ## indicated the non-significance with LO and HER while it exhibited the significance with other treatments and saline water (*p* < 0.05).

The anticancer activity of LO, HER, CS-LO-PEG NPs, and CS-LO-PEG HER-NPs was tested in vivo BT474 (breast cancer) tumor-bearing BALB/c female mice model (Figure 6a,b). The tumor volume and body weight were measured at different time intervals up to the end of the experiment. The tumor growth rapidly increased in the saline water treated (control group) ~558.25 ± 18.25 mm^3^ at the end of the experiment. In contrast, LO, HER, or nanoparticles treated groups exhibited promising antitumor proliferation activity (Figure 6c,d). Moreover, the level of the tumor growth did not show any significance between the LO and HER treatments; however, the CS-LO-PEG HER-NPs exhibited significant tumor growth inhibition as compared to other treatments, including the control group (Figure 6c,d). Next, the bodyweight changes between the treatments were measured, and the results displayed significant difference between the CS-LO-PEG HER-NPs treatments and the control group (saline water), while other treatments did not show any significant differences from the control (*p* < 0.05). Also, the experiment observed a 100% survival rate in all the groups at the end of the experiment.

#### 3.3.2. Histopathology

**Figure 7:**
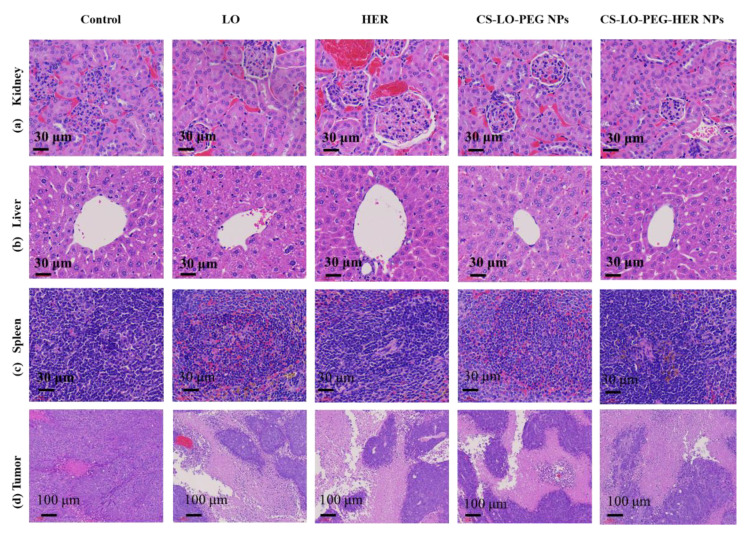
Effect of L-lysine α-oxidase, herceptin, and different nanoparticles treatment on histopathological changes in organs and tumor pathology. Kidney (**a**), liver (**b**), spleen (**c**), tumor (**d**). Where CS is chitosan, LO is L-lysine α-oxidase, PEG is polyethylene glycol 600, HER is herceptin (Trastuzumab).

The effect of LO, HER, CS-LO-PEG NPs, and CS-LO-PEG HER-NPs treatments was observed by H and E staining in histopathological changes in the major organs such as kidney, liver, and spleen (Figure 7a–c). There was no apparent damage observed in the representative sections of the kidney, liver, and spleen when compared between the control and various treatments. These results also indicated mild histological changes in the HER (anti-HER2+) treated group, while all other drug formulations did not cause significant damage to the kidney, liver, and spleen. Furthermore, the present results evidenced the biocompatibility of drug formulations in vivo at the tested concentration. The nanomedicine-mediated tumor cell necrosis or apoptosis is often studied by the H and E staining assay [57]. Therefore, the changes in tumor cells were observed by H and E staining. As shown in Figure 7d, no apparent necrosis was observed in the saline water treated group while the necrosis was observed in the sample treated mice group in the decreasing order of CS-LO-PEG HER-NPs > CS-LO-PEG NPs > LO > HER. Similar observations of the non-necrosis in tumors treated with saline water has been reported [58]. Moreover, the density of the tumor tissue was found to be higher in the control group than in other treatments, while the CS-LO-PEG HER-NPs displayed a significant reduction of tumor cells. Overall, the in vivo anticancer experiment demonstrated that pH-responsive, HER2+ targeting property, CS-LO-PEG HER-NPs enhanced the utilization of LO efficiently to achieve a promising anticancer effect. 

## 4. Conclusions

In summary, the CS-LO-PEG NPs were prepared by the ionic gelation self-assembly method, and further functionalized with anti-HER2+ by covalent bonding. The formulation of these nanoparticles was confirmed by physicochemical characterization using FTIR, NMR, TEM, and zeta potential particle size analysis. The dual functions of CS-LO-PEG HER-NPs (i) pH-responsive release; and (ii) targeting the HER2+ breast cancer cells (BT474) were confirmed by comparing them to other nanoparticles used in the study. The cellular internalization of CS-LO-PEG HER-NPs was confirmed by TEM. It also increased the controlled release of LO at the cancer site by sensing the pH of the tumor site. Finally, this work proved that CS-LO-PEG HER-NPs exhibited significant anticancer potency in BT474-xenograft tumor mice, with promising biocompatibility as indicated by the experiments of cytotoxicity, hemolysis, and organ toxicity. 

## Figures and Tables

**Table 1:** Effect of different concentrations of enzyme input on size, zeta potential, polydispersity index (PDI), drug entrapment and drug loading in chitosan-PEGylated nanoparticles (CS-LO-PEG NPs). The results are presented as mean ± SE. Superscript letters on the values indicates significant differences between the data in the column (*p* < 0.05).

Initial Enzyme Input (mg)	Size (nm)	Zeta Potential (mV)	PDI	Enzyme Entrapment (%)	Enzyme Loading (%)
0.1	138.53 ± 4.68 ^a^	26.73 ± 1.02 ^a^	0.35 ± 0.04 ^c^	75.77 ± 0.76 ^d^	5.26 ± 0.31 ^a^
0.2	143.76 ± 2.43 ^b^	31.10 ± 0.92 ^b^	0.44 ± 0.01 ^d^	62.82 ± 0.82 ^c^	10.59 ± 0.64 ^b^
0.5	144.1 ± 2.96 ^b^	35.86 ± 1.33 ^c^	0.28 ± 0.03 ^b^	59.15 ± 0.44 ^b^	14.26 ± 0.85 ^c^
1	182.60 ± 2.34 ^c^	36.60 ± 0.30 ^d^	0.12 ± 0.05 ^a^	53.38 ± 0.57 ^a^	17.49 ± 0.81 ^d^

## Data Availability

Not applicable.

## Supplementary Materials

The following supporting information can be downloaded at https://www.mdpi.com/article/10.3390/pharmaceutics14050927/s1: Figure S1. Determination of size (a,c,e) and zeta potential (b,d,f) of the nanoparticles. Z-average size and zeta potential of CS-LO NPs (a,b), CS-LO-PEG NPs (b–d) and CS-LO-PEG-HER NPs (e,f). Where CS is chitosan, LO is L-lysine α-oxidase, PEG is Polyethylene glycol 600, HER is Herceptin (Trastuzumab); Figure S2. Pictorial visualization of hemolysis activity of the nanoparticles CS-LO-PEG NPs and CS-LO PEG-HER NPs.

## References

[1] Kusakabe H., Kodama K., Kuninaka A., Yoshino H., Misono H., Soda K. (1980). A new antitumor enzyme, L-lysine alpha-oxidase from Trichoderma viride. Purification and enzymological properties. J. Biol. Chem..

[2] Lukasheva E.V., Berezov T.T. (2002). L-Lysine α-Oxidase: Physicochemical and Biological Properties. Biochemistry.

[3] Lukasheva E.V., Babayeva G., Karshieva S.S., Zhdanov D.D., Pokrovsky V.S. (2021). L-Lysine α-Oxidase: Enzyme with Anticancer Properties. Pharmaceuticals.

[4] Amano M., Mizuguchi H., Sano T., Kondo H., Shinyashiki K., Inagaki J., Tamura T., Kawaguchi T., Kusakabe H., Imada K. (2015). Recombinant expression, molecular characterization and crystal structure of antitumor enzyme, L-lysine α-oxidase from Trichoderma viride. J. Biochem..

[5] Pokrovsky V.S., Treshalina H.M., Lukasheva E.V., Sedakova L.A., Medentzev A.G., Arinbasarova A.Y., Berezov T.T. (2013). Enzymatic properties and anticancer activity of L-lysine α-oxidase from Trichoderma cf. aureoviride Rifai BKMF-4268D. Anti.-Cancer Drugs.

[6] Kusakabe H., Kodama K., Kuninaka A., Yoshino H., Soda K. (1980). Effect ofl-Lysine α-Oxidase on Growth of Mouse Leukemic Cells. Agric. Biol. Chem..

[7] Griffith R.S., Norins A.L., Kagan C. (1978). A Multicentered Study of Lysine Therapy in Herpes simplex Infection. Dermatology.

[8] Lukasheva E.V., Makletsova M.G., Lukashev A.N., Babayeva G., Arinbasarova A.Y., Medentsev A.G. (2020). Fungal Enzyme l-Lysine α-Oxidase Affects the Amino Acid Metabolism in the Brain and Decreases the Polyamine Level. Pharmaceuticals.

[9] Molina M.A., Codony-Servat J., Albanell J., Rojo F., Arribas J., Baselga J. (2001). Trastuzumab (herceptin), a humanized anti-Her2 receptor monoclonal antibody, inhibits basal and activated Her2 ectodomain cleavage in breast cancer cells. Cancer Res..

[10] Saravanakumar K., Sathiyaseelan A., Mariadoss A.V.A., Jeevithan E., Hu X., Shin S., Wang M.-H. (2020). Dual stimuli-responsive release of aptamer AS1411 decorated erlotinib loaded chitosan nanoparticles for non-small-cell lung carcinoma therapy. Carbohydr. Polym..

[11] Lee S.J., Min H.S., Ku S.H., Son S., Kwon I.C., Kim S.H., Kim K. (2014). Tumor-targeting glycol chitosan nanoparticles as a platform delivery carrier in cancer diagnosis and therapy. Nanomedicine.

[12] Nascimento A.V., Singh A., Bousbaa H., Ferreira D., Sarmento B., Amiji M.M. (2016). Overcoming cisplatin resistance in non-small cell lung cancer with Mad2 silencing siRNA delivered systemically using EGFR-targeted chitosan nanoparticles. Acta Biomater..

[13] Schellmann N., Deckert P.M., Bachran D., Fuchs H., Bachran C. (2010). Targeted enzyme prodrug therapies. Mini-Rev. Med. Chem..

[14] Appel E., Rabinkov A., Neeman M., Kohen F., Mirelman D. (2010). Conjugates of daidzein-alliinase as a targeted pro-drug enzyme system against ovarian carcinoma. J. Drug Target..

[15] Majd M.H., Asgari D., Barar J., Valizadeh H., Kafil V., Coukos G., Omidi Y. (2013). Specific targeting of cancer cells by multifunctional mitoxantrone-conjugated magnetic nanoparticles. J. Drug Target..

[16] Kumar A., Zhang X., Liang X.-J. (2013). Gold nanoparticles: Emerging paradigm for targeted drug delivery system. Biotechnol. Adv..

[17] Singh R., Lillard J.W. (2009). Nanoparticle-based targeted drug delivery. Exp. Mol. Pathol..

[18] Baharifar H., Khoobi M., Bidgoli S.A., Amani A. (2019). Preparation of PEG-grafted chitosan/streptokinase nanoparticles to improve biological half-life and reduce immunogenicity of the enzyme. Int. J. Biol. Macromol..

[19] Singh A.P., Biswas A., Shukla A., Maiti P. (2019). Targeted therapy in chronic diseases using nanomaterial-based drug delivery vehicles. Signal Transduct. Target. Ther..

[20] Yuan S., Hua J., Zhou Y., Ding Y., Hu Y. (2017). Doxorubicin Loaded Chitosan-W18 O49 Hybrid Nanoparticles for Combined Photothermal-Chemotherapy. Macromol. Biosci..

[21] Kumar S., Jana A.K., Dhamija I., Maiti M. (2013). Chitosan-assisted immobilization of serratiopeptidase on magnetic nanoparticles, characterization and its target delivery. J. Drug Target..

[22] Sathiyaseelan A., Saravanakumar K., Mariadoss A.V.A., Wang M.-H. (2021). pH-controlled nucleolin targeted release of dual drug from chitosan-gold based aptamer functionalized nano drug delivery system for improved glioblastoma treatment. Carbohydr. Polym..

[23] Calvo P., Remuñán-López C., Vila-Jato J.L., Alonso M.J. (1997). Novel hydrophilic chitosan-polyethylene oxide nanoparticles as protein carriers. J. Appl. Polym. Sci..

[24] Scheeren L.E., Nogueira D.R., Macedo L.B., Vinardell M.P., Mitjans M., Infante M.R., Rolim C.M.B. (2016). PEGylated and poloxamer-modified chitosan nanoparticles incorporating a lysine-based surfactant for pH-triggered doxorubicin release. Colloids Surfaces B Biointerfaces.

[25] Dziawer Ł., Majkowska-Pilip A., Gaweł D., Godlewska M., Pruszyński M., Jastrzębski J., Wąs B., Bilewicz A. (2019). Trastuzumab-Modified Gold Nanoparticles Labeled with 211At as a Prospective Tool for Local Treatment of HER2-Positive Breast Cancer. Nanomaterials.

[26] Arinbasarova A.Y., Ashin V.V., Makrushin K.V., Medentsev A.G., Lukasheva E.V., Berezov T.T. (2012). Isolation and properties of L-lysine-α-oxidase from the fungus Trichoderma cf. aureoviride RIFAI VKM F-4268D. Microbiology.

[27] Yoon H.Y., Son S., Lee S.J., Gil You D., Yhee J.Y., Park J.H., Swierczewska M., Lee S., Kwon I.C., Kim S.H. (2014). Glycol chitosan nanoparticles as specialized cancer therapeutic vehicles: Sequential delivery of doxorubicin and Bcl-2 siRNA. Sci. Rep..

[28] Saravanakumar K., Mariadoss A.V.A., Sathiyaseelan A., Wang M.-H. (2020). Synthesis and characterization of nano-chitosan capped gold nanoparticles with multifunctional bioactive properties. Int. J. Biol. Macromol..

[29] Saravanakumar K., Sriram B., Sathiyaseelan A., Hu X., Mariadoss A.V.A., MubarakAli D., Wang M.-H. (2020). Molecular identification, volatile metabolites profiling, and bioactivities of an indigenous endophytic fungus (Diaporthe sp.). Process Biochem..

[30] Jaiswal Y., Tatke P., Gabhe S., Vaidya A. (2016). Antidiabetic activity of extracts of Anacardium occidentale Linn. leaves on n -streptozotocin diabetic rats. J. Tradit. Complement. Med..

[31] Sakhtianchi R., Atyabi F., Yousefpour P., Vasheghani-Farahani E., Movahedi A.-A.M., Dinarvand R. (2011). Targeted delivery of doxorubicin-utilizing chitosan nanoparticles surface-functionalized with anti-Her2 trastuzumab. Int. J. Nanomed..

[32] Niu S., Williams G.R., Wu J., Wu J., Zhang X., Chen X., Li S., Jiao J., Zhu L.-M. (2019). A chitosan-based cascade-responsive drug delivery system for triple-negative breast cancer therapy. J. Nanobiotechnology.

[33] Mandal B., Mittal N.K., Balabathula P., Thoma L.A., Wood G.C. (2015). Development and in vitro evaluation of core–shell type lipid–polymer hybrid nanoparticles for the delivery of erlotinib in non-small cell lung cancer. Eur. J. Pharm. Sci..

[34] Forest V., Pourchez J. (2017). Preferential binding of positive nanoparticles on cell membranes is due to electrostatic interactions: A too simplistic explanation that does not take into account the nanoparticle protein corona. Mater. Sci. Eng. C.

[35] Hu D., Pan M., Yang Y., Sun A., Chen Y., Yuan L., Huang K., Qu Y., He C., Wei Q. (2021). Trimodal Sono/Photoinduced Focal Therapy for Localized Prostate Cancer: Single-Drug-Based Nanosensitizer under Dual-Activation. Adv. Funct. Mater..

[36] Ito T., Sun L., Bevan M.A., Crooks R.M. (2004). Comparison of Nanoparticle Size and Electrophoretic Mobility Measurements Using a Carbon-Nanotube-Based Coulter Counter, Dynamic Light Scattering, Transmission Electron Microscopy, and Phase Analysis Light Scattering. Langmuir.

[37] Sathiyaseelan A., Saravanakumar K., Mariadoss A.V.A., Wang M.-H. (2020). Biocompatible fungal chitosan encapsulated phytogenic silver nanoparticles enhanced antidiabetic, antioxidant and antibacterial activity. Int. J. Biol. Macromol..

[38] Melo M.N., Pereira F.M., Rocha M.A., Ribeiro J.G., Diz F.M., Monteiro W.F., Ligabue R.A., Severino P., Fricks A.T. (2020). Immobilization and characterization of horseradish peroxidase into chitosan and chitosan/PEG nanoparticles: A comparative study. Process Biochem..

[39] Barth A. (2007). Infrared spectroscopy of proteins. Biochim. Biophys. Acta Bioenerg..

[40] Cipolatti E.P., Valério A., Nicoletti G., Theilacker E., Araújo P.H.H., Sayer C., Ninow J.L., de Oliveira D. (2014). Immobilization of Candida antarctica lipase B on PEGylated poly(urea-urethane) nanoparticles by step miniemulsion polymerization. J. Mol. Catal. B Enzym..

[41] Garg N.K., Dwivedi P., Campbell C., Tyagi R.K. (2012). Site specific/targeted delivery of gemcitabine through anisamide anchored chitosan/poly ethylene glycol nanoparticles: An improved understanding of lung cancer therapeutic intervention. Eur. J. Pharm. Sci..

[42] Antoniou J., Liu F., Majeed H., Qi J., Yokoyama W., Zhong F. (2015). Physicochemical and morphological properties of size-controlled chitosan–tripolyphosphate nanoparticles. Colloids Surfaces A Physicochem. Eng. Asp..

[43] Niu S.-J.C.C.-C., Kuo C.-F.H.S.-M. (2007). Evaluation of chitosan-g-PEG copolymer for cell anti-adhesion application. J. Med. Biol. Eng..

[44] Helmi O., Elshishiny F., Mamdouh W. (2021). Targeted doxorubicin delivery and release within breast cancer environment using PEGylated chitosan nanoparticles labeled with monoclonal antibodies. Int. J. Biol. Macromol..

[45] Yuan Z., Ye Y., Gao F., Yuan H., Lan M., Lou K., Wang W. (2013). Chitosan-graft-β-cyclodextrin nanoparticles as a carrier for controlled drug release. Int. J. Pharm..

[46] Gatenby R.A., Gillies R.J. (2004). Why do cancers have high aerobic glycolysis?. Nat. Rev. Cancer.

[47] Kesavan S., Meena K., Sharmili S.A., Govindarajan M., Alharbi N.S., Kadaikunnan S., Khaled J.M., Alobaidi A.S., Alanzi K.F., Vaseeharan B. (2021). Ulvan loaded graphene oxide nanoparticle fabricated with chitosan and d-mannose for targeted anticancer drug delivery. J. Drug Deliv. Sci. Technol..

[48] Wu S., Xu C., Zhu Y., Zheng L., Zhang L., Hu Y., Yu B., Wang Y., Xu F.-J. (2021). Biofilm-Sensitive Photodynamic Nanoparticles for Enhanced Penetration and Antibacterial Efficiency. Adv. Funct. Mater..

[49] Wang F., Li J., Tang X., Huang K., Chen L. (2020). Polyelectrolyte three layer nanoparticles of chitosan/dextran sulfate/chitosan for dual drug delivery. Colloids Surf. B Biointerfaces.

[50] Tai W., Mahato R., Cheng K. (2010). The role of HER2 in cancer therapy and targeted drug delivery. J. Control. Release.

[51] Accardo A., Aloj L., Aurilio M., Morelli G., Tesauro D. (2014). Receptor binding peptides for target-selective delivery of nanoparticles encapsulated drugs. Int. J. Nanomed..

[52] Master A.M., Gupta A.S. (2012). EGF receptor-targeted nanocarriers for enhanced cancer treatment. Nanomedicine.

[53] Ando T., Nagumo M., Ninomiya M., Tanaka K., Linhardt R.J., Koketsu M. (2018). Synthesis of coumarin derivatives and their cytoprotective effects on t -BHP-induced oxidative damage in HepG2 cells. Bioorganic Med. Chem. Lett..

[54] Pajaniradje S., Mohankumar K., Pamidimukkala R., Subramanian S., Rajagopalan R. (2014). Antiproliferative and Apoptotic Effects ofSesbania grandifloraLeaves in Human Cancer Cells. BioMed Res. Int..

[55] Murad H., Hawat M., Ekhtiar A., Aljapawe A., Abbas A., Darwish H., Sbenati O., Ghannam A. (2016). Induction of G1-phase cell cycle arrest and apoptosis pathway in MDA-MB-231 human breast cancer cells by sulfated polysaccharide extracted from Laurencia papillosa. Cancer Cell Int..

[56] Kuang W., Hu W., Ren H., Shao Y., Liu B. (2021). Plant derived coumestrol phytochemical targets human skin carcinoma cells by inducing mitochondrial-mediated apoptosis, cell cycle arrest, inhibition of cell migration and invasion and modulation of m-TOR/PI3K/AKT signalling pathway. Saudi J. Biol. Sci..

[57] Wu C., Wang C., Zheng Y., Zheng Y., Liu Z., Xu K., Zhong W. (2021). Triple Enzyme-Regulated Molecular Hydrogels for Carrier-Free Delivery of Lonidamine. Adv. Funct. Mater..

[58] Liao T., Liu C., Ren J., Chen H., Kuang Y., Jiang B., Chen J., Sun Z., Li C. (2021). A chitosan/mesoporous silica nanoparticle-based anticancer drug delivery system with a “tumor-triggered targeting” property. Int. J. Biol. Macromol..

