# Supplementary material for: Monoclonal Antibody Functionalized, and L-lysine α-Oxidase Loaded PEGylated-Chitosan Nanoparticle for HER2/Neu Targeted Breast Cancer Therapy
Source: Pharmaceutics. 2022 Apr 24;14(5):927. doi: 10.3390/pharmaceutics14050927 (PMC9146122; doi:10.3390/pharmaceutics14050927)
Supplement: Supplementary file 1 [file pharmaceutics-14-00927-s001.zip › pharmaceutics-1667578-supplementary.pdf]

<u>Supplementary information</u>: Monoclonal antibody functionalized, and L-lysine α-oxidase loaded PEGylated-chitosan nanoparticle for HER2/neu targeted breast cancer therapy

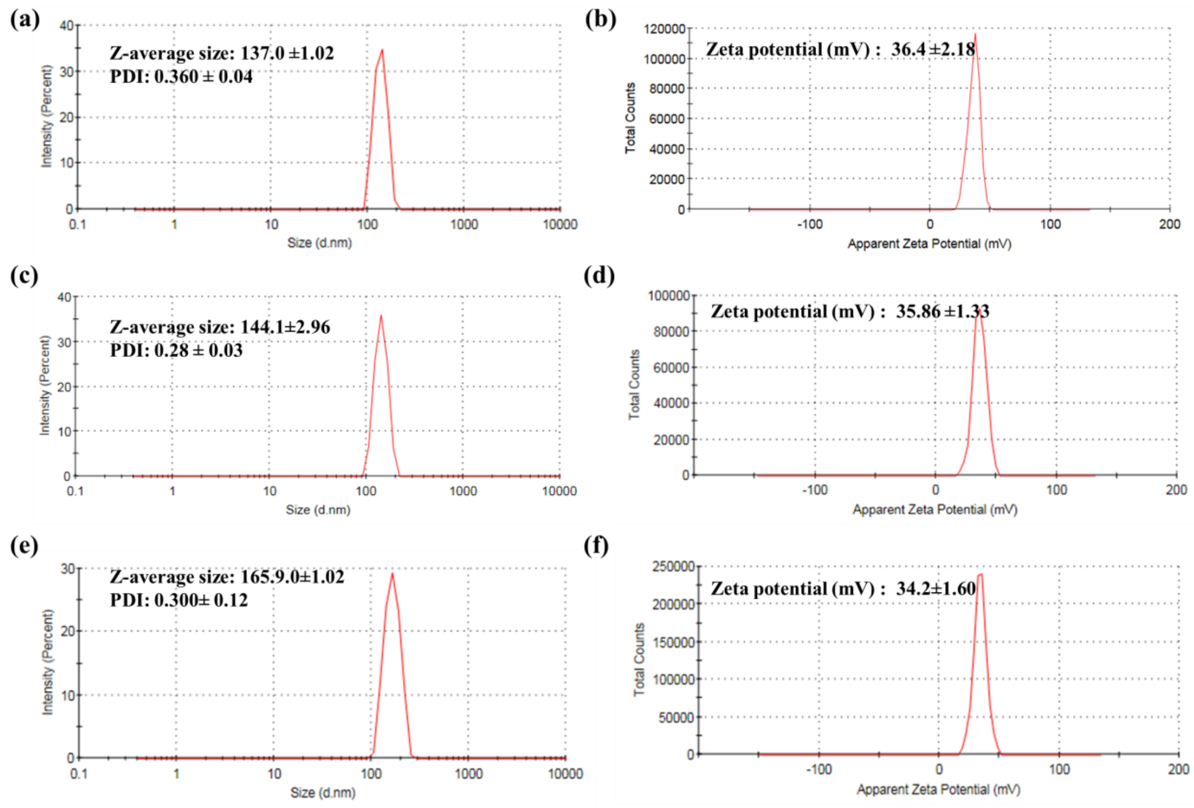

Fig.S1. Determination of size (a, c, e) and zeta potential (b, d, f) of the nanoparticles. Z-average size and zeta potential of CS-LO NPs (a, b), CS-LO-PEG NPs (c, d) (b) and CS-LO-PEG-HER NPs (e, f). where CS-chitosan, LO- L-lysine α-oxidase, PEG-Polyethylene glycol 600, HER-Herceptin (Trastuzumab).

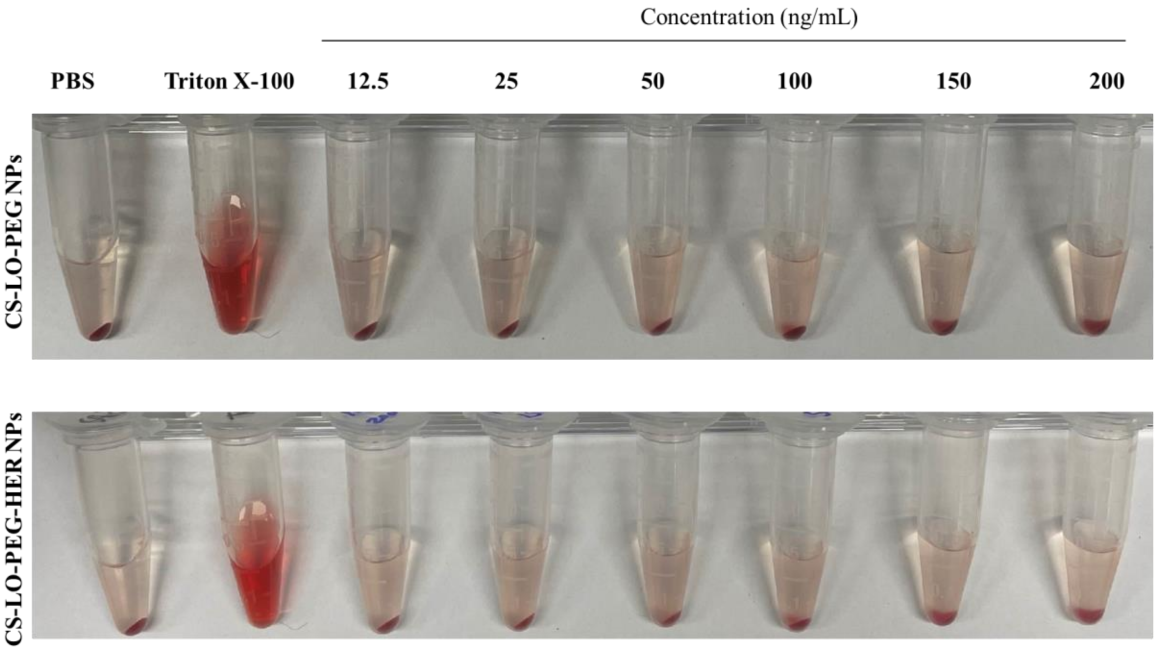

Fig. S2. Pictorial visualization of hemolysis activity of the nanoparticles CS-LO-PEG NPs and CS-LO PEG-HER NPs.
